# Ergosterol remodeling by the MARVEL protein Mrv6 links genotoxic stress adaptation to virulence in *Candida albicans*

**DOI:** 10.1016/j.jbc.2026.113414

**Published:** 2026-08-07

**Authors:** Wenxia Gao, Yi Zhou, Suna Jiang, Xingyi Tang, Jinrong Feng

**Affiliations:** Department of Pathogen Biology, School of Medicine, Nantong University, Nantong, Jiangsu, China

**Keywords:** *Candida albicans*, ergosterol, MARVEL domain, membrane permeability, virulence

## Abstract

How fungal pathogens coordinate genotoxic stress responses with membrane homeostasis to sustain cellular signaling remains poorly understood. In this study, we characterize Mrv6, a MARVEL domain-containing membrane protein that is prominently induced by genotoxic stress. Intriguingly, rather than functioning as a canonical DNA repair factor, Mrv6 acts as a critical biophysical adjuster governing membrane lipid homeostasis. Deletion of *MRV6* triggers a global transcriptional activation of the ergosterol biosynthetic pathway, resulting in aberrant cellular ergosterol accumulation. We demonstrate that this lipid imbalance fundamentally alters plasma membrane homeostasis, resulting in a less permeable architecture. Paradoxically, while this structural alteration inadvertently shields the fungus from exogenous genotoxins, conferring unexpected resistance to methyl methanesulfonate, it simultaneously hinders the intracellular transduction of environmental morphogenetic signals under hypoxic conditions. Consequently, the major hyphal repressor *NRG1* remains aberrantly expressed, leading to an attenuated signaling response that significantly impairs virulence. Crucially, targeted genetic suppression of either the lipid biosynthetic node (*ERG1*) or the downstream signaling node (*NRG1*) effectively mitigates this biophysical constraint, rescuing both morphogenetic switching and pathogenicity. Collectively, our findings define a novel biochemical paradigm wherein *Candida albicans* utilizes a stress-responsive membrane protein to fine-tune ergosterol remodeling and membrane permeability, structurally coupling stress adaptation to signal transduction.

*Candida albicans* is a commensal fungus commonly found in humans that can transition into an opportunistic pathogen, particularly in immunocompromised individuals (1). The pathogenicity of *C. albicans* relies on several key virulence traits, including its ability to switch from yeast form to invasive hyphal form for tissue penetration, strong adherence to host surfaces, formation of drug-resistant biofilms, and the secretion of tissue-damaging hydrolases like proteases and phospholipases (2, 3, 4). Emerging evidence underscores a close link between the DNA damage response (DDR) and fungal virulence (5). Indeed, disruption of critical DDR checkpoints, such as the kinases Rad53 and Mec1 or repair factors like Rad52 and Rtt109, has been shown to significantly impair virulence (6, 7). Therefore, elucidating the functions of DDR components is essential for a comprehensive understanding the pathogenesis of *C. albicans*.

Our previous work mapped the transcriptional response of this fungal pathogen to methyl methanesulfonate (MMS), providing insights into its DDR network (8). Interestingly, alongside canonical repair genes, we identified several upregulated genes encoding MARVEL domain-containing proteins, including *MRV8*, *MRV2*, and *MRV6*, that were strongly induced by exposure to MMS or hydroxyurea (HU) (9). The MARVEL domain is a structurally conserved four-transmembrane-helix motif first described in the myelin and lymphocyte protein family. Functionally, it typically associates with cholesterol-rich membrane regions to mediate essential cellular events such as vesicular trafficking and tight junction regulation, as demonstrated by MarvelD3 (10, 11). The critical role of MARVEL proteins is reflected in their broad involvement in complex diseases across species. In humans, the dysregulation of these proteins is closely linked to severe pathologies; for instance, the upregulation of MARVELD1 drives malignant phenotypes in glioma cells *via* the JAK/STAT signaling pathway (12, 13). Similarly, various CMTM family members (CMTM1, 2, 3, and 6) are associated with autoimmune conditions like rheumatoid arthritis, systemic sclerosis, Sjögren syndrome, and antiphospholipid syndrome (14). Beyond mammalian systems, MARVEL proteins also govern crucial processes in fungal pathogens. In the insect pathogen *Beauveria bassiana*, five different MARVEL proteins are required for full pathogenicity, coordinating nutrient utilization, stress tolerance, and dimorphic development, with *BbMARVEL2* specifically regulating conidial surface morphology (15).

In *C*. *albicans*, specific MARVEL proteins are known to regulate both morphogenesis and pathogenesis. For example, Nce102 coordinates actin organization and invasive growth, making it essential for virulence (16), while the deletion of *MRV8* severely impairs hyphal development, biofilm formation, and host epithelial damage (17). A unifying mechanistic feature of these membrane proteins is their strong preference for sterol-rich microdomains. In the model yeast *Saccharomyces cerevisiae*, Nce102 stably localizes to ergosterol-enriched regions, and its absence alters both the size and dynamics of vacuolar membranes (18). In pathogenic fungi, maintaining the integrity and proper organization of the plasma membrane is paramount. The membrane serves not only as a protective barrier against external stresses, but also acts as an active platform orchestrating key virulence traits, including cell wall synthesis, morphogenetic switching, endocytosis, and the secretion of virulence factors (19).

Despite these advances, how stress-induced MARVEL-domain proteins structurally and biochemically integrate with the distinct pathophysiology of *C. albicans* remains unclear. Among these, *MRV6* (*Orf19.3906*) is of particular interest due to its robust transcriptional induction by genotoxic stress and the absence of a clear ortholog in the non-pathogenic yeast *S. cerevisiae*. In this study, we reveal that Mrv6 functions not in classical DNA repair, but as a key regulator of ergosterol remodeling. We demonstrate that the loss of Mrv6 leads to an excessive accumulation of ergosterol that structurally alters the plasma membrane. Notably, this altered architecture creates a paradoxical biophysical constraint: while it inadvertently shields cells from exogenous DNA-damaging agents, it also impedes the intracellular entry of morphogenetic signals. Ultimately, our findings highlight a novel mechanistic link governing how *C. albicans* couples genotoxic stress adaptation to membrane homeostasis and signal modulation.

## Results

### *MRV6* is induced by genotoxic stressors in *C*. *albicans*

**Figure 1:**
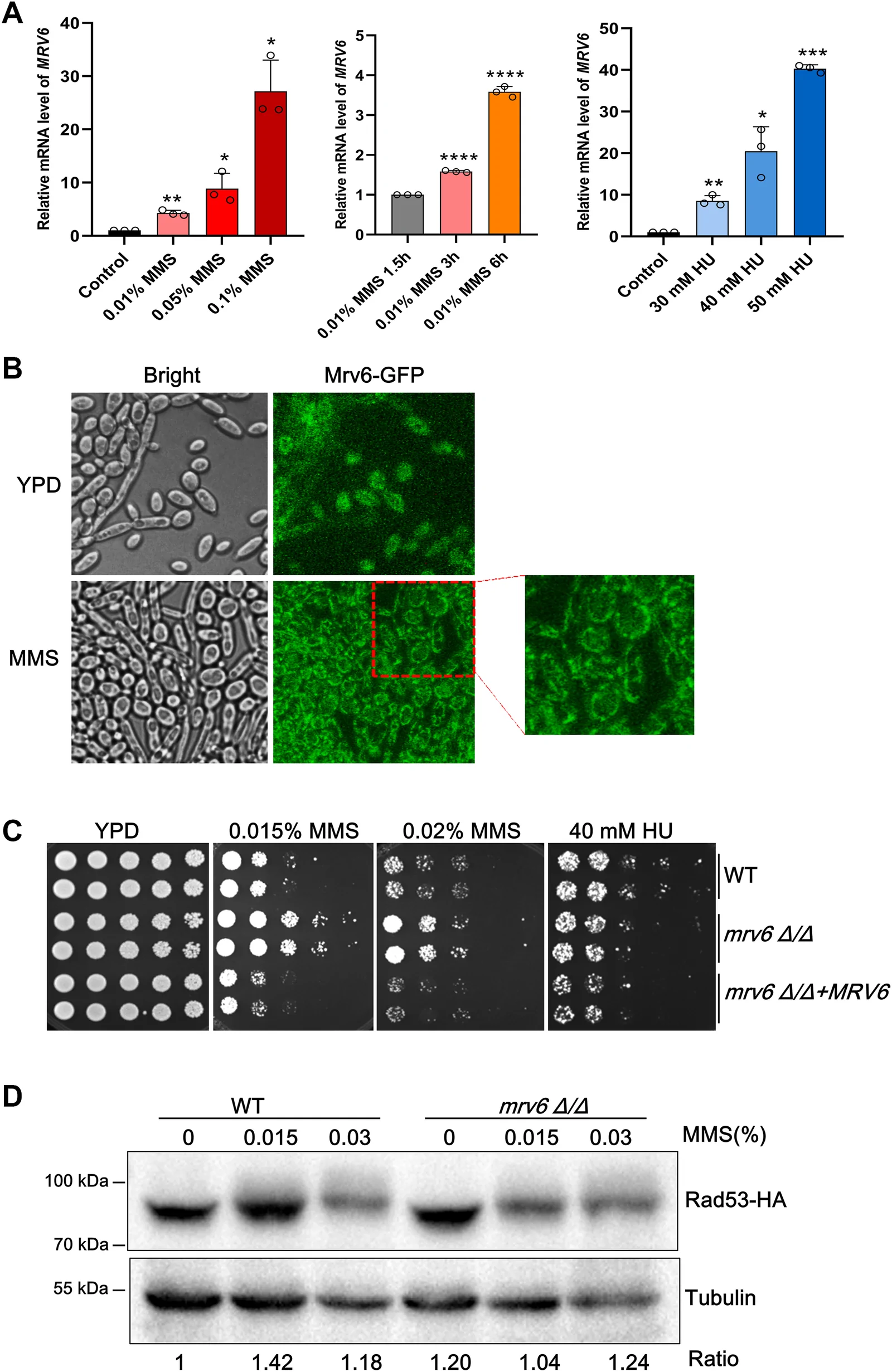
***MRV6* expression is induced by genotoxic stress in *C. albicans*.***A,* qRT-PCR analysis of *MRV6* transcript levels in response to MMS or hydroxyurea treatment. Log-phase WT cells were exposed to the indicated concentrations of stressors for 90 min *(left or right panel)* or for the specified durations *(middle panel)*. *B,* subcellular localization of Mrv6. WT cells expressing the Mrv6-GFP fusion protein were treated with or without 0.1% (v/v) MMS for 90 min prior to fluorescence microscopy imaging (400×). *C,* phenotypic assay of the *mrv6Δ* mutant under genotoxic stress. Data in *(A)* was presented as mean ± SD (n = 3). Statistical significance was determined using an unpaired two-tailed *t* test. *∗p* < 0.05, *∗∗p* < 0.01, *∗∗∗p* < 0.001, *∗∗∗∗p* < 0.0001. *D,* western blot analysis of Rad53. WT and *mrv6Δ* strains expressing Rad53-HA were treated with 0.015% and 0.03% MMS for 2 h. Untreated samples served as controls. The Rad53-HA to tubulin intensity ratio was compared. MMS, methyl methanesulfonate.

Our previous work identified *MRV6*, which encodes a MARVEL domain-containing protein, as a gene significantly upregulated under MMS stress (8). Given its predicted membrane localization, we sought to characterize its potential role in the DDR. In this study, we first analyzed the transcriptional dynamics of *MRV6* under MMS and HU exposure using qRT-PCR. Consistent with our RNA-seq data, a low dose of MMS (0.01%) induced a 3.3-fold increase in *MRV6* transcription relative to untreated controls (Fig. 1*A*). This induction followed a clear dose-dependent manner, with 0.05%- and 0.1%-MMS triggering 8.2-fold and 27.2-fold increases, respectively. Similarly, treatment with HU also led to dose-responsive transcriptional activation, with 50 mM HU eliciting the strongest induction.

Bioinformatic analysis predicted four transmembrane helices in Mrv6, suggesting it is an integral membrane protein. To experimentally confirm this, we generated a strain expressing a C-terminally GFP-tagged Mrv6 fusion protein. Fluorescence microscopy showed that Mrv6-GFP predominantly localizes to the plasma membrane under both basal conditions and MMS stress, with additional localization observed in intracellular vesicles (Fig. 1*B*).

To investigate the cellular function of Mrv6, we generated a homozygous deletion mutant (*mrv6Δ/Δ*) and evaluated its phenotypic response to genotoxic stress. Interestingly, despite its strong transcriptional induction by MMS, the loss of *MRV6* conferred increased resistance to this agent (Fig. 1*C*). Complementation of the *mrv6Δ* strain restored MMS resistance to WT levels, confirming that *MRV6* negatively impacts MMS tolerance. In contrast, the *mrv6Δ* strain showed no altered sensitivity to the DNA replication stressor, HU.

To ascertain whether Mrv6 functionally intersects with the classical DDR signaling, we investigated the expression and phosphorylation status of Rad53, a central DDR checkpoint kinase. Immunoblot analysis using strains expressing HA-tagged Rad53 revealed no significant differences in either baseline or MMS-induced Rad53 protein levels between the WT and the *mrv6Δ* mutant. Furthermore, the characteristic electrophoretic mobility shift, indicative of Rad53 hyperphosphorylation, was comparably induced in both strains (Fig. 1*D*). These results imply that rather than operating as a core factor within the canonical DDR cascade, Mrv6 likely utilizes an alternative mechanism to counteract genotoxic stress.

### Deletion of MRV6 attenuates the virulence of C. albicans

**Figure 2:**
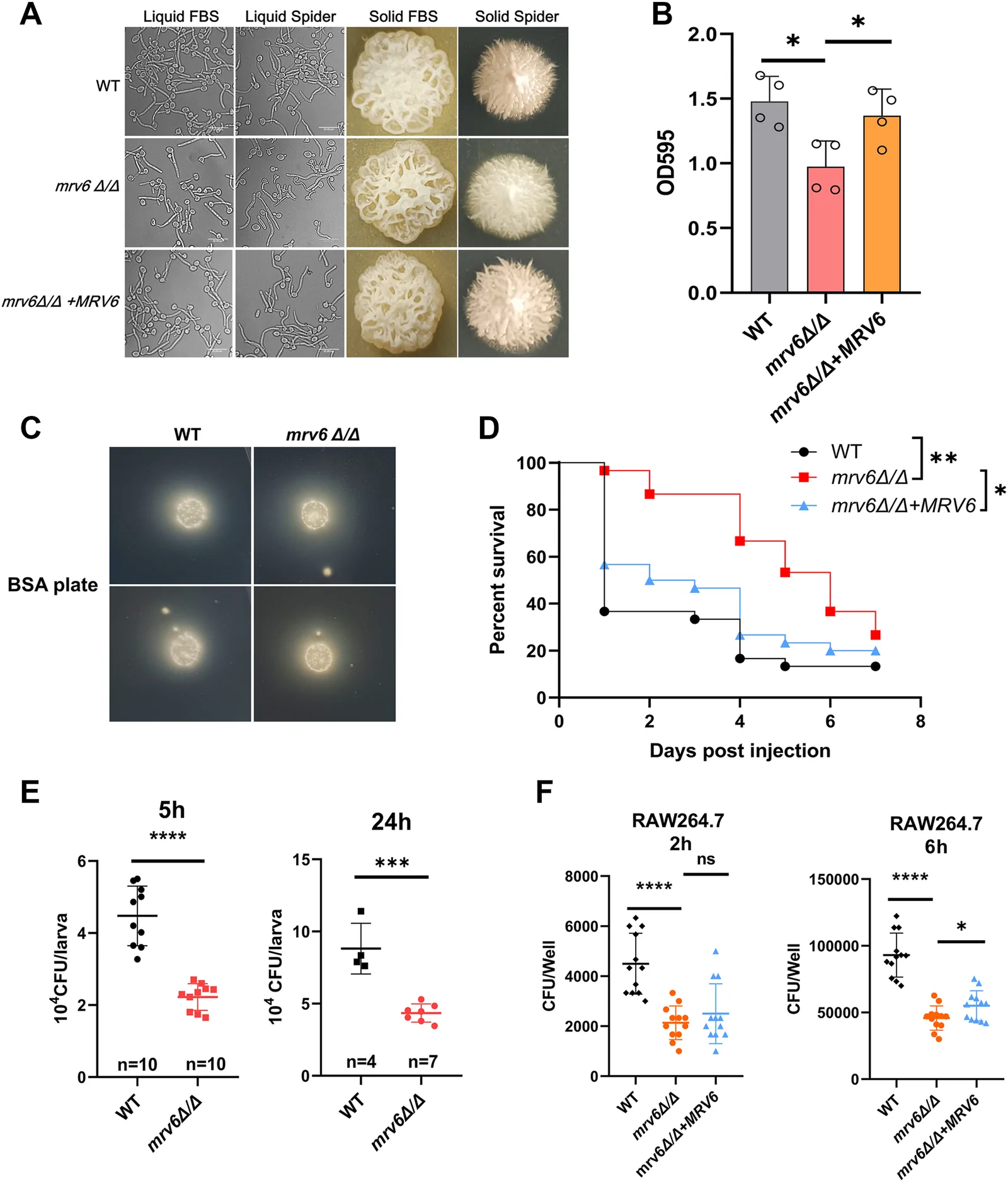
***MRV6* is required for the full virulence of *C. albicans*.***A,* hyphal formation assay. The indicated strains were cultured in liquid YPD medium supplemented with 20% FBS or Spider medium for 2 h at 37 °C. For hyphal formation on solid media, the strains were grown on solid YPD plus 20% FBS or Spider agar for 4 to 5 days at 37 °C. *B,* biofilm formation assay of the *mrv6Δ* strain (n = 4). *C,* extracellular protease activity. Indicated strains were spotted onto BSA agar plates and incubated at 37 °C for 2 to 3 days *D,* survival curves of *G. mellonella* larvae infected with WT, *mrv6Δ* mutant, or the complemented strain. Data from two independent colonies were pooled (n = 30 larvae per group). Survival data were analyzed using the Kaplan-Meier method and Log-rank test *via* GraphPad Prism 8.0.1 software. *∗p < 0.05, ∗∗p < 0.01*. *E,* fungal burden in infected larvae. Larvae were homogenized at the indicated time points post-infection to determine colony-forming units. *F,* macrophage interaction and fungal survival. RAW 264.7 macrophages were coincubated with fungal cells at an multiplicity of infection of 1:1. After 2 or 6 h of phagocytosis, internalized fungi were recovered *via* 0.01% SDS lysis, serially diluted, and plated for colony-forming unit counting. Quantitative data are presented as mean ± SD (n = 12), analyzed *via* unpaired two-tailed *t* test. *∗p* < 0.05, *∗∗p* < 0.01, *∗∗∗p* < 0.001, *∗∗∗∗p* < 0.0001. FBS, fetal bovine serum; YPD, yeast extract peptone dextrose.

Given the critical link between plasma membrane integrity and fungal pathogenesis, we investigated whether *MRV6* contributes to the virulence of *C. albicans*. We first evaluated hyphal formation in liquid Spider medium and fetal bovine serum (FBS)-supplemented media, observing no significant differences; the WT, *mrv6Δ* mutant and complemented strains all showed comparable hyphal morphology (Fig. 2*A*). Colony morphology on solid FBS or Spider media was similarly unchanged; all three strains formed wrinkled, irregular colonies that were indistinguishable from one another. We also assessed biofilm formation, which is crucial for evading host immunity and for antimicrobial resistance. Notably, deletion of *MRV6* led to a modest reduction in biofilm biomass, which decreased to about 65.5% of the WT level, and this defect was fully restored by reintroducing the gene (Fig. 2*B*). In addition, proteolytic activity assays on BSA-containing media revealed no obvious differences, with both WT and the *mrv6Δ* mutant producing similar halo zones around colonies (Fig. 2*C*).

To directly assess virulence, we employed a *Galleria mellonella* systemic infection model, which is a cost-effective, rapid, and ethically accessible *in vivo* system widely used for studying fungal virulence. While WT-infected larvae showed only 13.3% survival with a median survival time of 1 day, the *mrv6Δ* mutant group displayed significantly attenuated virulence, with the median survival time prolonged to 6 days (Fig. 2*D*). Reintroducing *MRV6* into the deletion strain fully restored virulence to WT levels, confirming that the gene contributes to pathogenic potential. Consistent with the survival data, quantitative determination of the internal fungal burden revealed a substantial proliferation defect for the mutant *in vivo*. At 5 h post-infection, WT-infected larvae carried approximately 4.5 × 10^4^ CFUs per larva, compared to only 2.2 × 10^4^ CFUs per larva in those infected with the *mrv6Δ* strain (Fig. 2*E*). Similarly, by 24 h post-infection, the WT group reached about 8.8 × 10^4^ CFUs per larva, whereas the *mrv6Δ* group yielded only 4.3 × 10^4^ CFUs per larva.

To further substantiate the effect of *MRV6* deletion on virulence, we investigated fungal survival within murine RAW 264.7 macrophage cells. Following a 2-h incubation with murine macrophages, the WT group yielded a CFU count of 4.5 × 10^4^/well. Notably, the CFU count for the *mrv6Δ* mutant group decreased to 2.1 × 10^4^/well, while the *MRV6*-complemented strain partially restored the CFU levels toward those of the WT strain (Fig. 2*F*). Similarly, after 6-h incubation, the *mrv6Δ* mutant group consistently exhibited lower CFU counts than the WT strain. Collectively, these data demonstrate that while *MRV6* appears to dispensable for hyphal development and proteolytic activity, it is required for robust biofilm formation and optimal proliferation within a host environment, ultimately contributing to the virulence of *C. albicans*.

### Transcriptional profiling of the *MRV6* deletion strain

**Figure 3:**
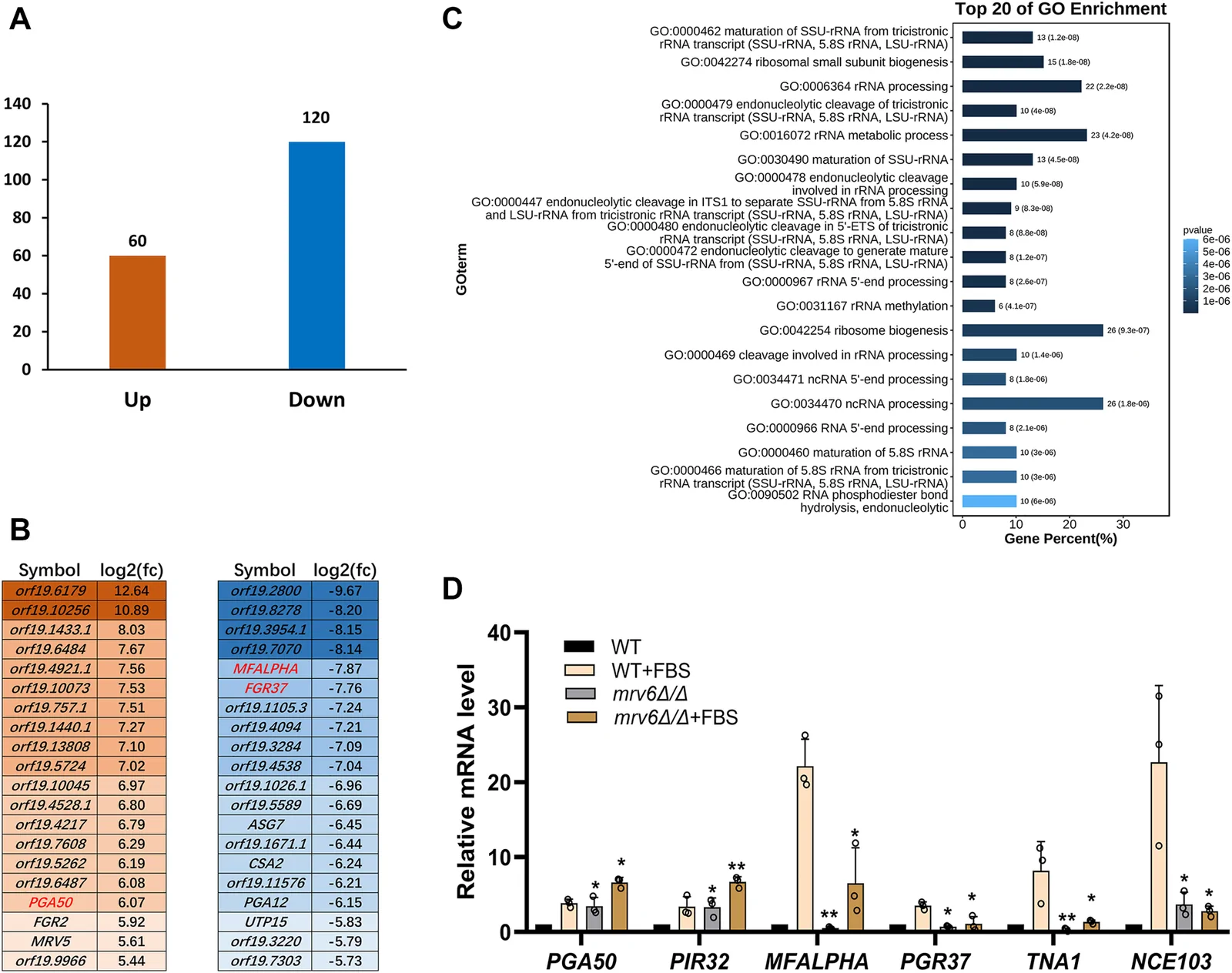
**Transcriptomic landscape of the *mrv6Δ* mutant.***A,* global transcriptional changes resulting from *MRV6* deletion. *B,* heatmap showing the *top* 20 most significantly DEGs. The Log_2_ fold change for each gene was listed. *C,* gene ontology enrichment analysis of the DEGs. The *top* 20 enriched gene ontology pathways are displayed. *D,* qRT-PCR validation of representative DEGs. Transcript levels in the *mrv6*Δ mutant were normalized to those in the WT strain under standard YPD conditions and following a 90-min induction with FBS. Data are presented as mean ± SD (n = 3). Statistical differences were evaluated by an unpaired two-tailed *t* test. *∗p* < 0.05, *∗∗p* < 0.01. DEG, differentially expressed gene; FBS, fetal bovine serum; YPD, yeast extract peptone dextrose.

To gain a global perspective on the cellular function of Mrv6, we performed RNA-seq to analyze the transcriptomic landscape of the *mrv6Δ* mutant. The loss of *MRV6* resulted in relatively modest transcriptional shifts, with 60 genes significantly upregulated and 120 downregulated (|log_2_ FC| > 1) (Fig. 3*A*, Table S2). Among the most upregulated transcripts were several uncharacterized open reading frame (ORFs), such as *orf19.6179* (12.64-fold), alongside *PGA50* (encoding a putative GPI-anchored protein) and *FGR2* (encoding a low-affinity phosphate transporter) (Fig. 3*B*). Conversely, the downregulated cohort was characterized by *MFALPHA* (encoding the alpha-factor mating pheromone, log_2_ FC = −7.87), *FGR37* (a potential filamentous growth regulator, log_2_ FC = −7.76), and several additional uncharacterized ORFs.

Gene Ontology enrichment analysis indicated that these differentially expressed genes were primarily involved in ribosome biogenesis, rRNA metabolic processes, and rRNA processing (Fig. 3*C*), hinting at a potential link between Mrv6 and ribosomal activity. To validate these findings, we performed qRT-PCR on representative genes under both basal and FBS-inducing conditions. Consistent with the RNA-seq data, *PGA50* and *PIR32* were significantly upregulated regardless of the growth condition (Fig. 3*D*); specifically, *PGA50* transcript levels in the mutant were 3.4-fold higher than WT under basal conditions and 6.6-fold higher upon FBS induction. Conversely, *MFALPHA*, *PGR37*, *TNA1*, and *NCE103* all exhibited significant downregulation under FBS induction. Notably, *MFALPHA* levels dropped to 52.3% and 29.5% of WT levels under basal and FBS-induced conditions, respectively. Collectively, these qRT-PCR results corroborate the global transcriptional changes observed in the *MRV6*-deficient strain, hinting at a broad role for Mrv6 in driving transcriptional reprogramming.

### Mrv6 negatively regulates ergosterol biosynthesis

**Figure 4:**
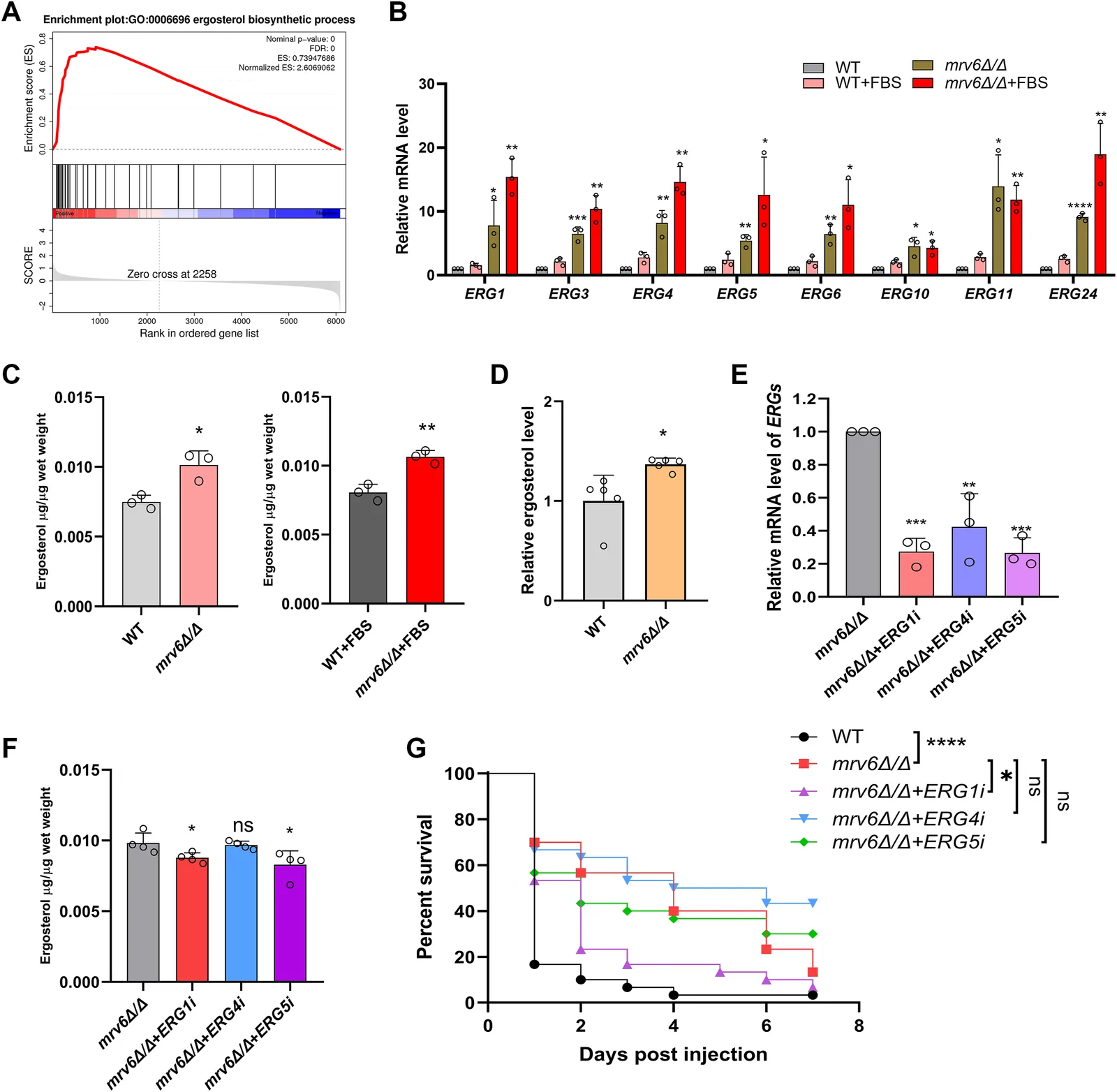
**Mrv6 negatively regulates the ergosterol biosynthesis pathway.***A,* gene set enrichment analysis of the *mrv6*Δ transcriptome. Ergosterol biosynthetic process was enriched. *B,* qRT-PCR quantification of *ERG* gene transcription. Expression levels in the *mrv6Δ* mutant were compared to the WT strain under YPD and FBS-inducing conditions (90 min) (n = 3). *C,* total cellular ergosterol content measurement. WT and *mrv6Δ* strains collected in YPD or following 2-h FBS induction were used for ergosterol analysis (n = 3). *D,* relative quantification of intracellular ergosterol by LC-MS. The integrated peak areas obtained from the specific multiple reaction monitoring transition were normalized against the wet cell weight (n = 5). *E,* qRT-PCR validation (n = 3) and *F,* corresponding ergosterol level measurement for CRISPRi-mediated repression of *ERG1*, *ERG4*, and *ERG5* in the *mrv6*Δ genetic background (n = 4). *G,* virulence assay in the *G. mellonella* infection model. The *mrv6*Δ mutant carrying the dCas9-mediated repression cassette for *ERG1, ERG4* or *ERG5* were used (n = 30 larvae per group). Quantitative analyses utilized unpaired two-tailed t-tests or Log-rank tests (for survival). *∗p < 0.05, ∗∗p < 0.01, ∗∗∗∗p < 0.0001.* FBS, fetal bovine serum; YPD, yeast extract peptone dextrose.

To elucidate the molecular basis of the paradoxical phenotypes exhibited by the *mrv6Δ* mutant—heightened stress resistance paired with attenuated virulence, we systematically evaluated its transcriptomic profile using gene set enrichment analysis. We observed a striking enrichment of biological processes driving ergosterol biosynthesis (Fig. 4*A*), identifying Mrv6 as a critical negative regulator of fungal sterol homeostasis. Specifically, deletion of *MRV6* led to transcriptional upregulation of key ergosterol biosynthetic genes (*ERG3*, *ERG4*, *ERG5*), which encode enzymes required for the terminal steps of sterol synthesis. Consistent with this, qRT-PCR analysis confirmed a broad upregulation of *ERG* genes in the *mrv6Δ* mutant, both in standard yeast extract peptone dextrose (YPD) medium and under FBS-inducing conditions (Fig. 4*B*). In YPD medium, the transcriptional increases ranged from 4.5-fold (*ERG10*) to 13.9-fold (*ERG11*). Notably, FBS induction further amplified this effect, with all tested *ERG* genes displaying over 10-fold upregulation, indicative of a marked elevation in ergosterol biosynthetic activity. To determine if these transcriptional changes translated to metabolic alterations, we quantified total cellular ergosterol levels (Fig. 4*C*). Under standard YPD conditions, the ergosterol content in the *mrv6Δ* mutant was elevated to 0.0101 μg/μg (wet weight), compared to 0.0074 μg/μg in WT strain. A similar upregulation of ergosterol was also observed in the mutant under FBS-inducing conditions. To confirm the effect of *MRV6* deletion on sterol metabolism, we quantified the relative intracellular ergosterol levels *via* LC-MS. After normalizing the peak areas to the respective wet cell weights, the *mrv6Δ* mutant showed an increased ergosterol level compared with the WT strain (Fig. 4*D*), which is consistent with the result generated from spectrophotometric method.

Given that *MRV6* deletion induces ergosterol accumulation, we hypothesized that this ergosterol remodeling might contribute to the attenuated virulence of the mutant. To test this, we employed a CRISPR interference (CRISPRi/dCas9) system to repress the transcription of specific *ERG* genes in the *mrv6Δ* background. qRT-PCR verified that *ERG1* expression reduced to 27.3% of the control level, and both *ERG4* and *ERG5* were also significantly downregulated (Fig. 4*E*). Accordingly, cellular ergosterol levels were effectively reduced in the *mrv6Δ* strains carrying either the *ERG1* or *ERG5* knockdown cassettes (Fig. 4*F*). Finally, we evaluated the impact of repressing *ERG* expression on fungal virulence using a *G*. *mellonella* infection model (Fig. 4*G*). Notably, suppressing *ERG1* in the *mrv6Δ* strain significantly reversed its virulence defect, reducing the host median survival time from 4 days to 2 days, which is comparable to that of the WT strain. Suppression of *ERG5* also trended toward reversing the virulence defect, albeit without statistical significance, whereas *ERG4* repression had no observable impact. Taken together, these results demonstrate that Mrv6 is required for maintaining ergosterol homeostasis in *C. albicans*, and the remodeling of ergosterol upon *MRV6* deletion compromises fungal virulence.

### Mrv6 is required for hyphal development under hypoxic conditions

**Figure 5:**
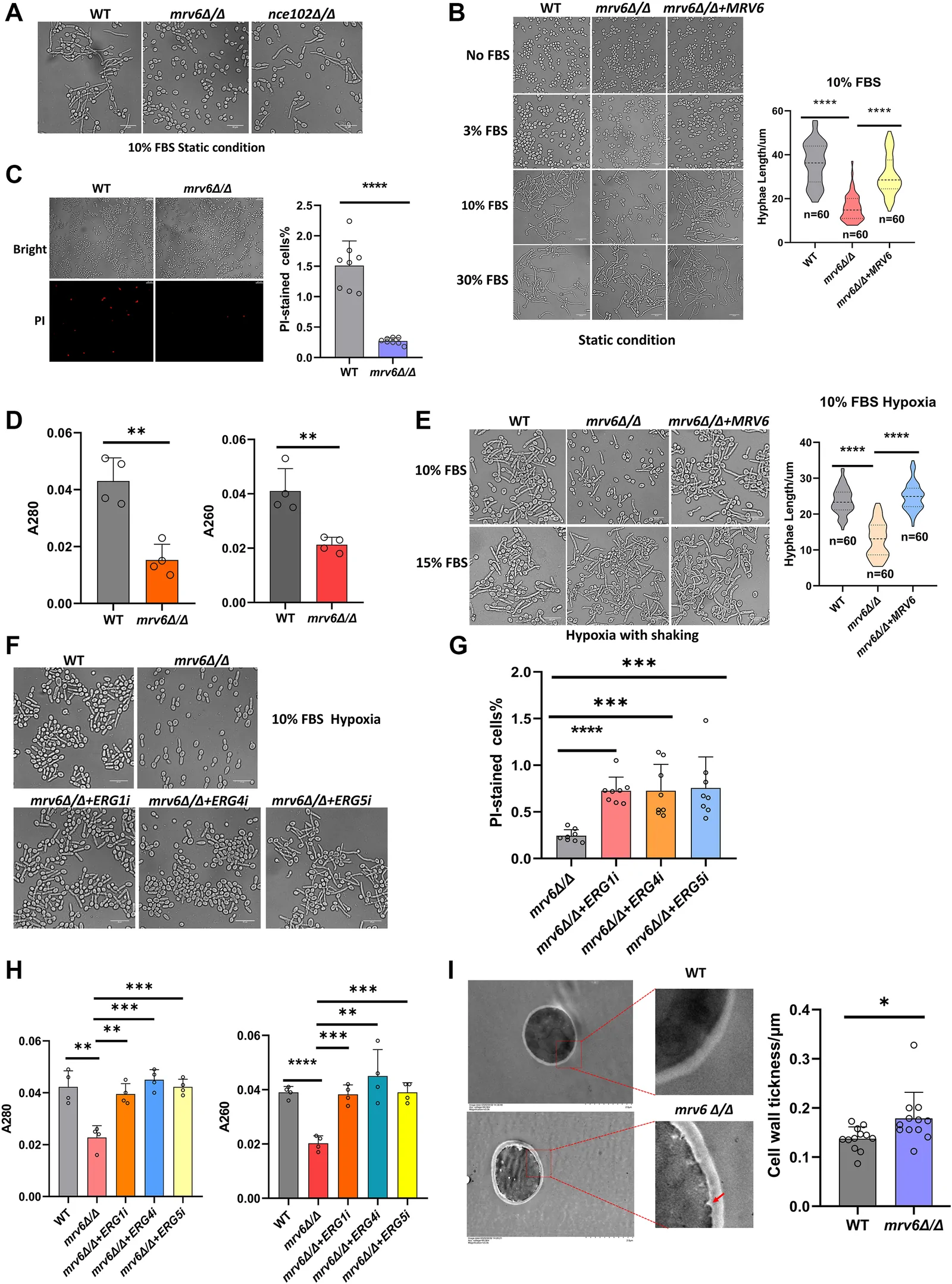
***MRV6* regulates hyphal development under static conditions.***A and B,* hyphal formation of the *mrv6Δ* mutant under static conditions. In *panel A*, a *nce102 Δ* mutant was used as a control. The indicated strains were kept in 10% FBS media under static condition. In *panel B*, the dose-dependent effect of FBS on hyphal induction was assessed. Hyphal length quantification is shown on the *right*. *C* and *G,* cell membrane permeability analysis using PI staining. The PI-positive cells in each group were counted and compared (n = 8). *D* and *H,* cell membrane leakage assay. The release of intracellular proteins and nucleic acids was spectrophotometrically determined at 280 nm and 260 nm, respectively (n = 4). *E* and *F,* hyphal formation analysis under hypoxia. The indicated strains were kept in 10% or 15% FBS media under hypoxia condition, with length quantification on the *right*. The *mrv6*Δ mutant carrying the dCas9-mediated repression cassette for *ERG1, ERG4* or *ERG5* were used in *panel F*. *I,* transmission electron microscopy analysis of cell ultrastructure. Overnight cells of WT and *mrv6Δ* mutant strains were checked by transmission electron microscopy. *Redarrows* indicate morphological alterations in the cell membrane. Quantification of cell wall thickness is displayed on the *right*. Quantitative data are presented as mean ± SD (n = 12), analyzed *via* unpaired two-tailed *t* test. *∗∗p* < 0.01, *∗∗∗∗p* < 0.0001. The scale bar represents = 20 μm. FBS, fetal bovine serum.

The related MARVEL-domain protein Nce102 was previously shown to govern *C. albicans* filamentation uniquely under static or embedded environments (20). Given our finding that Mrv6 profoundly alters membrane homeostasis, we reasoned that this lipid-remodeling might mechanically constrain cell surface receptors, thereby exerting a comparable block on morphogenetic signaling. To test this, we evaluated hyphal formation of the *mrv6Δ* strain under static condition with FBS induction. Consistent with previous reports, the *nce102Δ* mutant in the SN152 background exhibited a filamentation defect, with fewer cells developing into mature hyphae (Fig. 5*A*). Notably, the *mrv6Δ* mutant displayed a similar defect in hyphal formation under these conditions.

We further explored this defect across a range of FBS concentrations. In the absence of FBS or at a low dose (3%), all strains (WT, *mrv6Δ* mutant, and the complemented strain) predominantly maintained yeast morphology with no significant difference (Fig. 5*B*). However, at 10% FBS, while WT cells developed robust hyphae (average length 36.1 μm), the *mrv6Δ* mutant exhibited impaired filamentation with significantly shorter germ tubes (average length 15.8 μm). Reintroduction of *MRV6* into the deletion strain fully restored WT-like hyphal morphology. Interestingly, at a high FBS concentration (30%), both WT and *mrv6Δ* strains formed normal hyphae, suggesting that high levels of inducing signals can bypass the requirement for Mrv6.

Given Mrv6's plasma membrane localization and the previously observed resistance to MMS, we hypothesized that *MRV6* deficiency might alter physical membrane properties. To test this, we used propidium iodide (PI) staining, which only enters cells with compromised membranes (Fig. 5*C*). While 1.51% of WT cells were PI-positive, the *mrv6Δ* mutant showed significantly lower uptake (0.27%), suggesting a reduction in membrane permeability. We confirmed this by measuring the leakage of intracellular components. In the *mrv6Δ* strain, the leakage of nucleic acid was significantly reduced to 35.5% of the WT level (Fig. 5*D*). Similarly, protein leakage decreased to 51.8% of that observed in the WT. These results consistently indicate that the loss of *MRV6* leads to a "tighter," less permeable plasma membrane.

Since static (nonshaking) conditions inherently reduce dissolved oxygen levels, we used a hypoxia chamber to determine whether the hyphal defect of the *mrv6Δ* mutant is oxygen-dependent. Even under shaking (200 rpm) in 10% FBS, the mutant failed to filament normally under hypoxia with 10% FBS (Fig. 5*E*), a defect that was again eliminated by increasing the FBS concentration to 15%.

Because our earlier data showed that *MRV6* deletion leads to ergosterol accumulation, we tested whether suppressing ergosterol biosynthesis could rescue this filamentation failure. Indeed, dCas9-mediated inhibition of *ERG1* or *ERG5* in the *mrv6Δ* background successfully restored hyphal development to levels comparable to the WT (Fig. 5*F*). To further investigate the physiological consequences of inhibiting these biosynthetic nodes, we evaluated membrane permeability using PI staining. Consistently, the suppression of *ERG1*, *ERG4* or *ERG5* in the *mrv6Δ* mutant successfully normalized cell membrane permeability (Fig. 5*G*). Furthermore, repressing these biosynthetic nodes effectively restored basal membrane function, returning the leakage rates of intracellular proteins and nucleic acids to WT levels (Fig. 5*H*).

Finally, we used transmission electron microscopy to examine the impact of these lipid alterations on cell ultrastructure. Compared to the smooth membrane of WT strain, the plasma membrane of the *mrv6Δ* mutant exhibited severe structural irregularities, with frequent undulations. In addition, the mutant exhibited a markedly thicker cell wall (average 0.18 μm) compared to the WT (average 0.14 μm) (Fig. 5*I*). Taken together, these data suggest that Mrv6 regulates membrane permeability and organization; its absence likely restricts the uptake of extracellular inducing signals, thereby impairing morphogenetic switching under specific stress conditions.

### Mrv6 regulates hyphal formation in a *NRG1-*dependent manner

**Figure 6:**
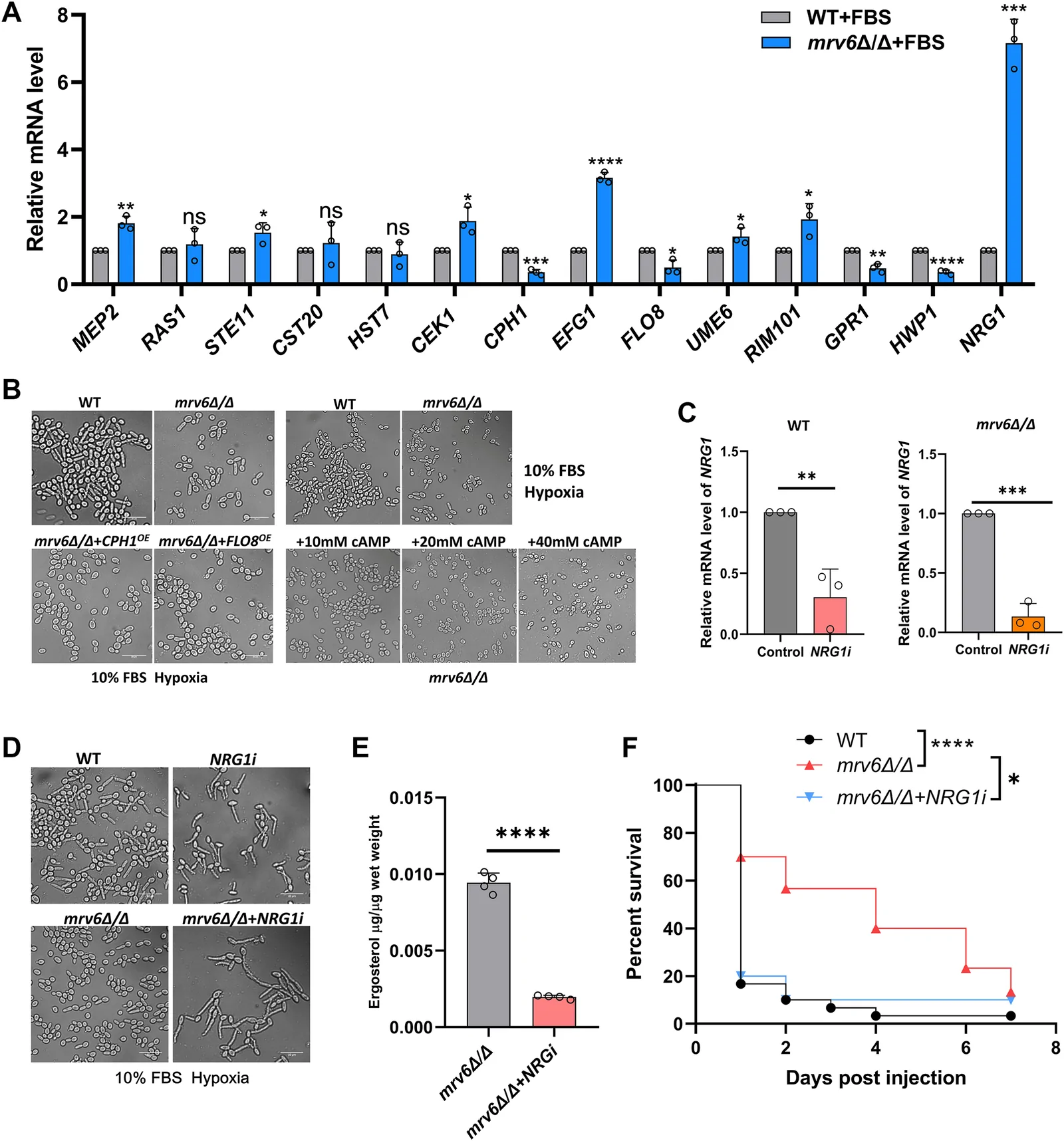
**Mrv6 governs hyphal morphogenesis *via* a *NRG1*-dependent pathway.***A,* qRT-PCR analysis of hyphal specific genes. WT and *mrv6Δ* mutant cells were induced with 10% FBS under hypoxia conditions (n = 3). *B,* effects of *FLO8* and *CPH1* overexpression or exogenous cAMP supplementation on hyphal restoration in the *mrv6*Δ mutant. *C* and *D,* characterization of *NRG1* repression in the *mrv6Δ* background, including qRT-PCR validation of *NRG1* knockdown *(C)* (n = 3) and its corresponding effect on hyphal formation assays *(D)*. *E,* total cellular ergosterol content in the *mrv6Δ* mutant carrying a dCas9-mediated *NRG1* repression cassette (n = 4). *F,* functional virulence assay in the *G. mellonella* model using the *mrv6Δ* mutant expressing a dCas9-mediated *NRG1* repression cassette. Data from two independent colonies were pooled (n = 30 larvae/group). Data are means ± SD. Statistical parameters: unpaired two-tailed *t* test or Kaplan-Meier Log-rank test. *∗p* < 0.05, *∗∗p* < 0.01, *∗∗∗p <* 0.001, *∗∗∗∗p* < 0.0001. The scale bar represents 20 μm. FBS, fetal bovine serum.

Hyphal formation in *C. albicans* is driven by various stimuli through two primary signaling pathways, cAMP-PKA and MAPK, which converge on the transcription factors Efg1 and Cph1. To investigate how Mrv6 modulates hyphal formation at the molecular level, we used qRT-PCR to examine the expression of hyphal-specific genes (HSGs) associated with these pathways under hypoxic conditions. In the *mrv6Δ* mutant, canonical HSGs exhibited transcriptional dysregulation (Fig. 6*A*). *CPH1*, a key downstream effector of the MAPK pathway, was expressed at only 35.3% of the WT level. In contrast, its upstream regulators, including *MEP2*, *RAS1*, *STE11*, *CST20*, and *CEK1*, showed no significant changes or even slight upregulation (*MEP2*, *STE11*, and *CEK1*). Other key regulators, such as *FLO8* and *GPR1*, exhibited varying degrees of downregulation, dropping to 49.7% and 48.0% of WT levels, respectively. However, *EFG1*, *UME6*, and *RIM101* were actually upregulated. Furthermore, the transcription of *HWP1*, which encodes a classic hyphal-specific protein, decreased to 35.3% of the WT level. Conversely, the transcription of *NRG1*, a major negative regulator of hyphal formation, increased 7.2-fold compared to the WT strain. Overall, the deletion of *MRV6* significantly alters the transcription of multiple HSGs and impairs hyphal development under hypoxia.

Since *CPH1* and *FLO8*, two key transcription factors regulating hyphal formation, were downregulated, we overexpressed each of these genes in the *mrv6Δ* mutant and tested its ability to form hyphae. However, the mutant carrying either the *CPH1* or *FLO8* overexpression cassette showed no obvious difference from the empty mutant (Fig. 6*B*). In addition, we suspect that cAMP-PKA signaling pathway may be suppressed in the mutant, so we added extra cAMP to the medium to boost the signal. But even with 40 mM cAMP, we did not observe a clear rescue of the hyphal formation defect in the *mrv6Δ* mutant (Fig. 6*B*).

Given the significant upregulation of *NRG1*, we replaced its promoter with a *MET3* promoter to directly modulate its transcription. qRT-PCR confirmed that this promoter replacement significantly downregulated *NRG1* expression in both the WT and *mrv6Δ* strains, even without supplemental methionine and cysteine (Fig. 6*C*). We then used these strains to evaluate hyphal formation in standard FBS media under hypoxic conditions. While the WT strain filamented normally, the *mrv6Δ* mutant showed a clear defect. Suppressing *NRG1* in the WT background slightly enhanced hyphal formation, with most cells developing normal hyphae. Notably, suppressing *NRG1* in the *mrv6Δ* strain successfully rescued its filamentation defect, resulting in hyphae that were even longer than those of the WT strain (Fig. 6*D*). Intriguingly, further investigation uncovered a reciprocal regulatory link between this morphogenetic signaling node and lipid homeostasis. Ergosterol quantification revealed that suppressing *NRG1* in the *mrv6Δ* background effectively mitigated the excessive ergosterol accumulation, reducing its cellular content to approximately 20.8% of that observed in the original mutant (Fig. 6*E*).

Finally, we investigated the effect of *NRG1* inhibition on the virulence of the *mrv6Δ* strain. While the *mrv6Δ* mutant alone showed clear virulence defect, suppressing *NRG1* in the mutant effectively restored its virulence to WT levels (Fig. 6*F*). Together, these findings indicate that Mrv6 regulates hyphal formation and virulence in a *NRG1*-dependent manner.

## Discussion

The DDR is a conserved cellular safeguard that transcends simple genomic repair, often involving the strategic reprogramming of global metabolism (21, 22, 23). In this study, we characterize Mrv6, a MARVEL domain-containing protein in *C*. *albicans* that exemplifies an unprecedented link between genotoxic stress adaptation and membrane homeostasis. Our work reveals that Mrv6 does not function as a canonical DNA repair factor but instead serves as a critical biochemical adjuster governing ergosterol homeostasis. The absence of Mrv6 leads to a significant ergosterol remodeling that alters the plasma membrane architecture, effectively uncoupling environmental sensing from morphogenetic signaling.

**Figure 7:**
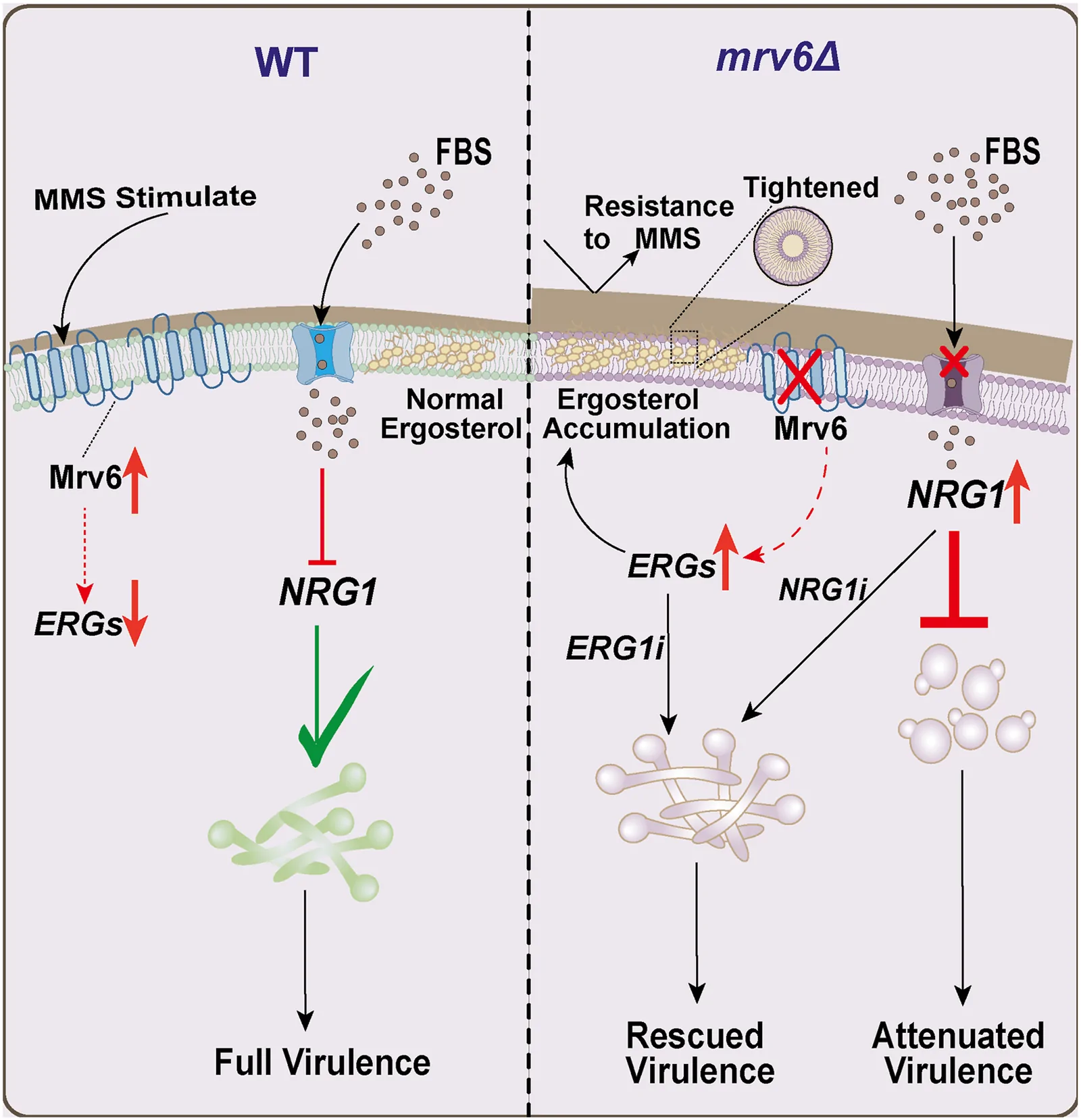
**Schematic model of the regulatory network governed by *MRV6* in *C. albicans*.** In WT cells *(left)*, Mrv6 localizes to the plasma membrane and maintains optimal ergosterol biosynthesis and normal membrane permeability, allowing efficient sensing of environmental cues to downregulate *NRG1* for morphogenetic switching. Loss of Mrv6 *(right)* triggers aberrant upregulation of *ERG* genes and ergosterol remodeling. This lipid imbalance physically "tightens" the plasma membrane and increases the thickness of cell wall (decreased permeability). The tightened membrane structure confers resistance to exogenous toxins, but effectively interferes the intracellular entry of morphogenetic signals. Consequently, the major hyphal repressor *NRG1* remains aberrantly expressed, primarily restricting the cells to a yeast-like state and attenuating overall virulence. Targeted inhibition of *ERG1* or *NRG1* relieves this physical and signaling constraints restoring fungal pathogenesis.

A central finding of our study is the Upc2-independent activation of the ergosterol biosynthetic pathway upon *MRV6* deletion. In fungi, the sterol-sensing transcription factor Upc2 is the master regulator of *ERG* gene expression. Intriguingly, we observed that *ERG* upregulation in the *mrv6Δ* mutant occurs despite a decrease in *UPC2* transcription and unaltered Upc2 nuclear localization (Fig. S2). This suggests the existence of a non-canonical, compensatory lipid-regulatory circuit. Given that MARVEL domains are structurally predisposed to partition into sterol-rich microdomains (lipid rafts), we propose that Mrv6 functions as a membrane-embedded sterol sensor. In WT cells, Mrv6 may facilitate a feedback loop that represses excessive sterol synthesis under stress (Fig. 7). This is supported by the inverse transcriptional relationship where *ERG* genes are downregulated precisely when *MRV6* is induced by MMS or HU (8, 9). The discovery of this Upc2-independent mechanism unveils a novel layer of lipid homeostasis regulation that is specifically tailored to the fungal stress response.

The metabolic consequence of this dysregulation, the aberrant accumulation of ergosterol, fundamentally alters the physical properties of the plasma membrane. Based on our permeability data, we propose a biophysical barrier model to explain the paradoxical phenotypes observed in the *mrv6Δ* mutant. Although further biophysical measurements (*e.g.,* membrane fluidity assays) are needed to fully understand these dynamics, our current findings strongly support this model. The increased lipid packing density results in a significantly "tightened" and less permeable membrane architecture. This functional barrier non-specifically restricts the influx of exogenous molecules, providing a plausible mechanism for the increased resistance to the alkylating agent MMS. Unlike a previous reported MMS resistance generated by deleting *GST1* (24), which relies on enhanced DNA repair (*e.g.,* the *GST1*-*RAD7* axis), the resistance in the *mrv6Δ* strain appears to be a passive, biophysical defense. While this ergosterol remodeling confers a survival advantage under pharmacological stress, it imposes a severe physical constraint on essential cellular signaling.

This signaling interference is most evident during hyphal morphogenesis under hypoxic conditions. We found that the filamentation defect in the *mrv6Δ* mutant is highly sensitive to inducer concentrations (FBS), suggesting a dose-dependent bypass of the membrane barrier. At low inducer levels, the "tightened" membrane prevents sufficient morphogenetic signals from reaching their receptors or being internalized. Consistent with this model, transcriptional analysis of hyphae-specific genes in the mutant revealed a dysregulated profile: *CPH1* and *FLO8* were downregulated, *EFG1* and *UME6* were upregulated, while *HST7* remained largely unchanged. Notably, overexpressing downstream transcription factors like *CPH1* or *FLO8* failed to rescue the defect, whereas the targeted suppression of the central repressor *NRG1* in the *MRV6* deletion strain effectively restored filamentation and also reduced its ergosterol level. This suggests that the morphogenetic failure is not due to a collapse of the core filamentous machinery, but rather a failure to relieve Nrg1-mediated repression due to a sensing blockade at the membrane interface.

The physiological consequences of this biochemical defect manifest as severely attenuated virulence and impaired biofilm architecture. While previous studies have implicated other MARVEL-domain proteins, such as Nce102 (20) and Mrv8 (17), in *C. albicans* biofilm formation and pathogenicity, the underlying molecular mechanisms have largely remained elusive. Our study addresses this critical gap by tracing these macroscopic developmental defects directly to a fundamental ergosterol imbalance. In both the *G. mellonella* infection model and macrophages, the *mrv6Δ* mutant exhibits a paradoxical pathogenic state: the altered membrane architecture and cell wall confer an incidental tolerance to exogenous stressors, yet render the pathogen functionally "blind" to the host-derived morphogenetic cues required for tissue invasion. Ultimately, this complex integration of determinants illustrates that successful pathogenesis necessitates a critically calibrated state of membrane homeostasis, continuously tuned by Mrv6 to ensure the cell remains mechanically permissive to environmental signaling.

In conclusion, our study defines Mrv6 as a novel mechanosensory node that bridges the gap between the DDR and membrane biology (Fig. 7). By regulating ergosterol remodeling, Mrv6 ensures that the plasma membrane remains sufficiently permeable for environmental signal transduction. This work expands the functional boundaries of stress-responsive genes and highlights membrane biophysical properties as a tunable platform for controlling fungal pathogenesis.

## Experimental procedures

The *C*. *albicans* WT strain used in this study was SN152. Strains were routinely cultured at 30 °C in YPD medium (1% yeast extract, 2% peptone, 2% glucose) supplemented with 50 mg/L uridine. Transformants were selected on synthetic dextrose (SD) medium (0.67% yeast nitrogen base, 2% glucose, and requisite amino acid supplements) lacking the appropriate auxotrophic marker. Solid media were prepared by adding 2% agar. MMS was purchased from Meryer (M75317). Hydroxyurea (A600528) and all amino acids were purchased from Sangon Biotech. Primers used in this study were listed in Table S1.

### Real time PCR (qRT-PCR)

To quantify gene expression, overnight cultures of the indicated strains were diluted 1:10 into fresh YPD and grown at 30 °C for 3 h. Cultures were then treated with specified concentrations of MMS or HU for 90 min; untreated cultures served as controls. Total RNA was extracted using the RNA-easy Isolation Reagent (Vazyme, R701) following bead-beating homogenization. Genomic DNA was removed and reverse transcription was performed using the first Strand cDNA Synthesis Kit with gDNA-Wiper (Vazyme, R312). qRT-PCR was conducted on a real-time PCR system using the ChamQ SYBR qPCR Master Mix (Vazyme, Q311). Relative gene expression was calculated using the standard 2−ΔΔCt method, with transcript levels normalized to the housekeeping gene GAPDH. Data was presented as the mean of at least three independent biological replicates.

### DNA manipulation

Deletion of the *MRV6* gene was performed in the SN152 background using a transient CRISPR/Cas9 system (25). The Cas9 expression cassette and sgRNA cassettes were amplified from plasmid pV1093. The repair DNA template, containing a *HIS1* marker, was amplified from plasmid pFA-HA-HIS1. These three fragments, including the Cas9 cassette, sgRNA, and repair template, were co-transformed into SN152 cells *via* the lithium acetate method. Transformants were selected on SD plates lacking histidine, and successful deletion was confirmed by PCR (Fig. S1). A similar protocol was employed to delete *NCE102* in the SN152 back ground using *HIS1* as a selective marker (Fig. S1).

For phenotypic verification, the full-length *MRV6* locus, including its promoter, ORF, and terminator, was amplified from the genomic DNA. The pV1093-sgHIS1 plasmid was linearized by *Sac* I and *Kpn* I digestion. The linearized plasmid and the *MRV6* PCR product were cotransformed into the *mrv6Δ* mutant. Complemented strains were selected on YPD plates containing 200 μg/ml nourseothricin, and correct integration was verified by PCR.

To examine the cellular localization of Mrv6, a GFP tag was amplified from the pFA-GFP-ARG4 plasmid and integrated downstream of the *MRV6* ORF in SN152, thereby generating a Mrv6-GFP fusion protein. Correct integration was verified by PCR.

To achieve targeted transcriptional repression, a dCas9-mediated interference system was employed. Briefly, the interference plasmid targeting a given gene was constructed by amplifying pRS159 with a primer pair that incorporated the sgRNA sequence, thereby yielding a series of pRS159-sgRNA plasmids. The resulting sgRNA-carrying plasmid was then linearized using *Pac* I and introduced into the *MRV6* deletion strain. Transformants were isolated on YPD plates containing 200 μg/ml nourseothricin and subsequently subjected to spot assay and virulence analysis.

To overexpress *CPH1* or *FLO8*, we first generated a CIP10-ADH1-ARG4 plasmid by substituting the *URA3* marker in CIP10-ADH1-URA3 (8) with the *ARG4* marker. The entire ORF including the terminator, of either *CPH1* or *FLO8* was then cloned into the *Kpn* I and *Xho* I sites of the CIP10-ADH1-ARG4 plasmid, using the One Step Cloning Kit (10923ES50,). The resulting construct was linearized with *Stu* I and introduced into the *MRV6* deletion strain. To suppress *NRG1* transcription, we replaced its native promoter with the *MET3* promoter amplified from the pFA-MET3-ARG4 plasmid, using a transient CRISPR/Cas9 system as previously mentioned. Correct promoter replacement was confirmed by PCR, and *MRV6* transcription levels were verified by qRT-PCR.

### Protein extraction and western blot analysis

To evaluate the phosphorylation status of Rad53, an HA epitope tag was chromosomally integrated at the C-terminus of the *RAD53* locus in both the WT and the *mrv6Δ* strains. Exponentially growing cell cultures were exposed to 0.015% and 0.03% (v/v) MMS for 2 h prior to harvesting. The cell pellets were subsequently resuspended in a lysis buffer (P0013, Beyotime) supplemented with 10 mM PMSF and a phosphatase inhibitor cocktail (1:50, P1082, Beyotime). Following the addition of an equivalent volume of acid-washed glass beads, the cells were mechanically disrupted utilizing a disruptor (SI-DD68). This homogenization step comprised six cycles of 60 s agitation, interspersed with 2-min incubation intervals on ice.

The obtained crude lysates were clarified by centrifugation (12,000×*g*, 15 min, 4 °C), and total protein concentrations were determined using a Bradford protein assay kit (E112). Equal amounts of total protein (approximately 10 μg per lane) were resolved by 10% SDS-PAGE to separate the unphosphorylated and phosphorylated forms of Rad53. The separated proteins were then electroblotted onto polyvinylidene fluoride membranes at a constant current of 350 mA for 150 min in a standard transfer buffer (25 mM Tris, 192 mM glycine, 0.1% SDS, 20% [v/v] methanol). Following a 1-h blockade of non-specific binding sites with 5% (w/v) non-fat dry milk in TBS-T buffer (20 mM Tris-HCl, pH 7.6, 150 mM NaCl, 0.1% Tween 20), the membranes were subjected to overnight incubation with primary antibodies. Primary antibodies used included a mouse anti-HA monoclonal antibody (1:2500, Cat# AE008, ABclonal) to detect HA-tagged Rad53 and its phosphorylation-dependent mobility shifts, and a mouse anti-β-tubulin monoclonal antibody (1:2500, Cat# 66240, Proteintech) serving as the endogenous loading control. The specificity of the anti-HA antibody was internally validated by the absence of immunoreactive bands in lysates from a WT strain lacking the Rad53-HA cassette. Protein bands were developed using the Meilunbio fg-super Sensitive ECL luminescence reagent (MA0186-1, Meilunbio). Images were captured using a ChemiDoc Touch Imaging System (Bio-Rad), and densitometric analysis was performed using Image Lab software (version 55.2.1, Bio-Rad; https://www.bio-rad.com/en-us/product/image-lab-software).

### Fluorescence microscopy

WT cells expressing Mrv6-GFP were cultured in YPD medium at 30 °C to mid-log phase and then treated with 0.02% MMS for 90 min. Untreated cells were processed in parallel as controls. Cells were harvested, washed once with PBS, mounted on glass slides, and immediately examined under a Leica DM5000B fluorescence microscope using a 40× objective.

### PI staining

Overnight cultures were diluted 1:10 into fresh YPD medium and grown at 30 °C for 8 h to reach stationary phase. Cells were harvested by centrifugation, washed twice with ice-cold PBS, and resuspended in 0.1 M sodium citrate containing 10 U/ml lyticase (L02XG, Omega Bio-Tek). The cell suspension was incubated at 30 °C for 1 h with gentle agitation to remove the cell wall. The resulting protoplasts were pelleted, washed twice with PBS, and resuspended in 1 ml of PBS. PI solution was added to a final concentration of 2.5 μg/ml. After 10-min incubation in the dark at room temperature, the stained protoplasts were washed twice with PBS to remove excess dye. Samples were mounted on glass slides and observed using a Leica DM5000B fluorescence microscope (40× objective).

### Cell membrane leakage assay

Membrane permeability was evaluated by measuring the leakage of UV-absorbing intracellular components, following a previously described protocol (26). Briefly, overnight cultures of WT and *mrv6Δ* mutant cells were collected, washed twice, and resuspended in PBS to a final density of 2 × 10^7^ cells/ml (1 ml total volume). The suspensions were incubated at 30 °C with shaking for 4 h. Subsequently, the cell suspensions were centrifuged at 10,000 rpm for 10 min. The resulting supernatants were filtered through a 0.22 μm filter. The release of nucleic acids and proteins was quantified by measuring the absorbance of the filtered supernatant at 260 nm and 280 nm, respectively, using a NanoDrop UV-Vis spectrophotometer (Thermo Fisher Scientific).

### Hyphal formation assay

Hyphal induction was performed in liquid media using either 20% FBS or Spider medium (1% peptone, 1% mannitol, 0.2% K_2_HPO_4_). Overnight cultures were diluted 1:20 into liquid YPD containing 20% FBS or into Spider medium. Cultures were incubated at 37 °C with shaking for 2 h. Cell morphology was examined by microscopy and hyphal length was quantified using ImageJ (v2.14.0; https://imagej.nih.gov/ij/).

Hyphal morphogenesis on solid substrates was evaluated on YPD plates supplemented with 20% FBS or on Spider plates. Overnight cultures were serially diluted to 10^−4^ with distilled water and 2 μl aliquots were spotted onto YPD agar supplemented with 20% FBS or onto Spider agar plates. Plates were incubated at 37 °C for 3 to 5 days, and colony morphology was imaged daily.

To assess hyphal formation under static conditions, cells were inoculated at 1 × 10^6^ cells/ml in liquid media with varying FBS concentrations (0–30%) and incubated at 37 °C for 1.75 h without shaking (20). For hypoxic conditions, overnight cultures were diluted 1:20 in 10% FBS medium and placed in an anaerobic chamber with an anaerobic packet (C-02, MGC) to maintain oxygen concentration at approximately 6 to 12%. The chamber was incubated at 37 °C with shaking for 1 h, after which cells were collected and hyphal morphogenesis was examined as previously described. To test the role of cAMP in inducing hyphal formation, Dibutyryl-cAMP (MeilunBio) was added to 10% FBS medium at a final concentration of 10, 20 or 40 mM.

### Biofilm formation assay

Biofilm biomass was quantified using a crystal violet staining method, adapted from a previous protocol (23). Briefly, WT or *MRV6* deletion strains were cultured in SC medium at 30 °C with shaking for approximately 16 h. The cultures were adjusted to an OD595 of 1.0- and 1-mL aliquots were dispensed into 24-well polystyrene plates. Biofilms were formed by static incubation at 37 °C for 48 h. Non-adherent cells were removed by aspiration, and the wells were washed three times with 1 ml PBS per well. Biofilms were stained with 0.4% crystal violet for 45 min at room temperature. After extensive washing with distilled water, the bound dye was solubilized in 95% ethanol, and the absorbance was measured at 595 nm.

### Extracellular protease secretion assay

Proteolytic activity was assessed using a BSA-containing agar plate assay (27). Aliquots (2 μl) of tenfold-diluted overnight cultures were spotted onto BSA plates (0.01% amino acids, 0.1% KH_2_PO_4_, 0.05% MgSO_4_, 2% glucose, 0.2% BSA, 2% agar). Plates were incubated at 37 °C for 2 to 3 days to permit the development of transparent halos.

### Virulence studies

The *G. mellonella* larva model was used to assess virulence (28). Larvae (around 300 mg each) were injected into the last left proleg with 10 μl of a *C. albicans* cell suspension (2.5 × 10^7^ cells/ml). Infected larvae were maintained at 37 °C and survival was monitored daily. Mortality data for each strain were consolidated, and survival curves were plotted using the Kaplan-Meier method and analyzed with Prism 8 (GraphPad Software; https://www.graphpad.com). To assess *in vivo* fungal burden, infected larvae were collected at designated time points. Living larvae were surface-sterilized with 75% ethanol and then homogenized in 1 ml of PBS using an electric grinder. The resulting homogenates were diluted 10-fold and plated onto YPD agar supplemented with chloramphenicol (50 μg/ml) to inhibit bacterial growth. Plates were incubated at 30 °C for 2 days before CFU enumeration. Fungal burden was expressed as CFU per individual larva, determined from cohorts of 4 to 10 larvae per experimental group.

### Survival ability assay of C. albicans cells in macrophages

The murine RAW 264.7 macrophage cell line (obtained from the Cell Bank of the Chinese Academy of Sciences) was appropriately cultured and genetically authenticated *via* short tandem repeat profiling by Generalbiol. For the assays, the macrophages were seeded into 12-well plates at a density of 5 × 10^4^ cells per well. Macrophages were then challenged with *C. albicans* at a multiplicity of infection of 1:1. The cocultures were incubated at 37 °C in a humidified 5% CO_2_ atmosphere for two or 6 h. Following incubation, the wells were washed three times with PBS to remove non-adherent and extracellular fungal cells. To quantify the internalized fungi, macrophages were lysed with 500 μl of cold 0.01% SDS per well to release the internalized fungi. The lysates were serially diluted, plated onto YPD agar, and incubated at 30 °C for 48 h. Fungal survival was determined by counting the CFUs.

### RNA preparation and RNA-seq assay

Two independent colonies of WT and *MRV6* deletion strains were cultured in YPD medium at 30 °C with shaking (200 rpm). Overnight cultures were then diluted 1:20 in medium containing 20% FBS and incubated at 37 °C with shaking for 2 h. Total RNA was extracted using the TRIzol reagent (Invitrogen) according to the manufacturer’s instructions. RNA quality was assessed using an Agilent 2100 Bioanalyzer (Agilent Technologies) and confirmed by electrophoresis on RNase-free agarose gels. For library construction, eukaryotic mRNA was enriched from total RNA using Oligo(dT) magnetic beads and then fragmented into segments of 200 to 700 bp with a standardized fragmentation buffer. First-strand cDNA was synthesized using the NEBNext Ultra RNA Library Prep Kit for Illumina (NEB #7530; New England Biolabs, Ipswich), followed by second-strand synthesis to generate double-stranded cDNA. The resulting fragments were subjected to end repair, 3′ adenylation, and ligation of Illumina-compatible adapters. After purification with AMPure XP Beads (1.0× bead ratio) to remove unligated adapters and reaction components, the adapter-ligated DNA was amplified by PCR to generate the final sequencing library. Sequencing was performed on an Illumina NovaSeq 6000 platform by OmicsMaster Biotechnology Co., Ltd (, generating an average of approximately six gigabases of paired-end reads per sample. Reads were aligned to the *C. albicans* SC5314 reference genome (NCBI: GCF_000182965.3). Principal component analysis confirmed a clear transcriptomic separation between the WT and *MRV6* deletion groups. Differential gene expression was analyzed using DESeq2 (v1.20.0), with gene-wise dispersion parameters estimated internally *via* maximum likelihood approaches and parametric shrinkage (29). Furthermore, gene set enrichment analysis was applied to the expression matrices to identify significantly enriched GO terms and KEGG pathways. Pathways fulfilling the criteria of an absolute normalized enrichment score > 1, a nominal *p*-value < 0.05, and a false discovery rate q-value < 0.25 were considered defined as statistically significant. Raw and processed data are available in the National Genomics Data Center (NGDC) Genome Sequence Archive (GSA) under accession number CRA036175.

### Detection of ergosterol content

Wild-type and *mrv6Δ* mutant strains were initially grown overnight in YPD medium at 30 °C with shaking (200 rpm). These cultures were then diluted 1:10 into 10 ml of fresh YPD and incubated for an additional 3 h under the same conditions. For ergosterol assessment under hyphal-inducing conditions, overnight cultures were instead diluted 1:20 in medium containing 20% FBS and incubated at 37 °C with shaking for 2 h. Cells were harvested by centrifugation (3000 rpm, 5 min) and washed twice with sterile distilled water. Following a final centrifugation (10,000 rpm, 5 min), the supernatant was removed completely, and the net wet weight of the pellet was recorded.

Each pellet received 500 μl of 25% alcoholic potassium hydroxide solution (prepared by dissolving 2.5 g KOH in 3.5 ml sterile distilled water and bringing the volume to 10 ml with absolute ethanol), followed by vortex mixing for 1 min. The resulting suspensions were transferred into 5-mL tubes and heated in an 85 °C water bath for 1 h, with gentle mixing performed twice during the incubation. After cooling to room temperature, sterols were extracted by adding 200 μl of sterile distilled water and 600 μl of n-heptane, followed by vigorous vortexing for 3 min. The heptane layer was then collected into a fresh microcentrifuge tube and kept at −20 °C for 24 h (30).

An aliquot of 2 μl of the extract was measured between 240 and 300 nm using a NanoDrop spectrophotometer. The detection of ergosterol and the late sterol intermediate 24(28)-DHE gave rise to a characteristic four-peaked absorption curve. Ergosterol content was calculated as a percentage of the pellet wet weight using the following formulas:

$$\text{Ergosterol}+24\left(28\right)-\text{DHE}\%=\left[A_{281.5}/290\times F\right]/\text{pellet}\;\text{weight}$$

$$24\left(28\right)-\text{DHE}\%=\left[A_{230}/518\times F\right]/\text{pellet}\;\text{weight}$$

Here, F represents the dilution factor in ethanol, and 290 and 518 are the E values (percentage per centimeter) determined for crystalline ergosterol and 24(28)-DHE, respectively. Results are expressed as mean ± SEM from three independent replicates.

### LC-MS relative quantification of intracellular ergosterol

To confirm the change in intracellular ergosterol after deleting *MRV6*, LC-MS analysis was conducted. Following the previously described ergosterol extraction procedures, the dried samples were reconstituted in methanol and injected into an AB SCIEX Qtrap 5500 LC-MS system equipped with a Turbo Spray ionization source operating in the positive ion mode. Targeted quantification was performed using multiple reaction monitoring, specifically tracking the ergosterol precursor-to-product ion transition from m/z 379.3 to 69.1. To assess relative differences across experimental groups, the integrated peak areas derived from this specific multiple reaction monitoring transition were normalized to the initial wet cell weight of the corresponding biological replicates. Finally, relative ergosterol levels were expressed as fold changes, with the average value of the WT control arbitrarily set to 1.0.

### Transmission electron microscopy analysis

Overnight cultures (0.3 ml) of the wild-type and mrv6Δ mutant strains were harvested and initially fixed with 1 ml of 4% electron microscopy grade glutaraldehyde, then placed at 4 °C overnight. Following fixation, the cells were embedded in 100 μl of 30% agarose and immersed in 1 ml of 4% glutaraldehyde. Ultrathin sections were generated, double-stained with uranyl acetate and lead citrate, and examined using a transmission electron microscopy (JEOL Ltd).

## Data availability

The datasets generated and analyzed during the current study are available from the corresponding author upon reasonable request. The RNA-seq data have been deposited in the National Genomics Data Center (NGDC) Genome Sequence Archive (GSA) under accession number CRA036175.

## Supporting information

This article contains supporting information.

## Conflict of interest

The authors declare that they have no conflicts of interest with the contents of this article.
