# Supplementary material for: Ergosterol remodeling by the MARVEL protein Mrv6 links genotoxic stress adaptation to virulence in Candida albicans
Source: J Biol Chem. 2026 Aug 7;302(10):113414. doi: 10.1016/j.jbc.2026.113414 (PMC13570312; doi:10.1016/j.jbc.2026.113414)
Supplement: Supplementary file 1 [file mmc1.docx]

**Ergosterol remodeling by the MARVEL protein Mrv6 links genotoxic stress adaptation to virulence in *Candida albicans***

Wenxia Gao^1#^, Yi Zhou^1#^ , Suna Jiang^1^, Xingyi Tang^1^，Jinrong Feng^1*^

1 Department of Pathogen Biology, School of Medicine, Nantong University, Nantong, Jiangsu, China, 226006；

**Supporting information**

**Figure S1 PCR validation of *MRV6* and *NCE102* deletion strains.**

**Figure S2 The transcription and cellular localization of Upc2 in the *mrv6Δ* mutant.** (A) qRT-PCR analysis of *UPC2* transcription. WT and *mrv6Δ* mutant cells were induced with 10% FBS under hypoxia conditions. (B) Cellular localization of GFP‑Upc2. A GFP tag was integrated at the N‑terminus of Upc2.

**Table legends**

Table S1 Primers used in this study.

Table S2 Differentially expressed genes（DEGs）after deleting *MRV6*.
